# Using Comorbidity Statistical Modeling to Predict Inpatient Mortality: Insights Into the Burden on Hospitalized Patients

**DOI:** 10.7759/cureus.45899

**Published:** 2023-09-25

**Authors:** Hezborn M Magacha, Sheryl M Strasser, Shimini Zheng, Venkata Vedantam, Adedeji O Adenusi, Adegbile, Oluwatobi Emmanuel

**Affiliations:** 1 Internal Medicine, Quillen College of Medicine, East Tennessee State University, Johnson City, USA; 2 Public Health, Georgia State University, Atlanta, USA; 3 Biostatistics, College of Public Health, East Tennessee State University, Johnson City, USA; 4 Internal Medicine, Interfaith Medical Center, Brooklyn, USA; 5 Epidemiology and Biostatistics, College of Public Health, East Tennessee State University, Johnson City, USA

**Keywords:** inpatient mortality, predicting mortality, mortality, age-adjusted charlson comorbidity index, charlson comorbidity index

## Abstract

Background

The expenditures of the United States for healthcare are the highest in the world. Assessment of inpatient disease classifications associated with death can provide useful information for risk stratification, outcome prediction, and comparative analyses to understand the most resource-intensive chronic illnesses. This project aims to adapt a comorbidity index model to the National Inpatient Sample (NIS) database of 2020 to predict one-year mortality for patients admitted with select International Classification of Diseases, 10th Edition (ICD-10) codes of diagnoses.

Methodology

A retrospective cohort study analyzed mortality with comorbidity using the Charlson comorbidity index model (CCI) in a sample population of an estimated 5,533,477 adult inpatients (individuals aged ≥18 years) obtained from the National Inpatient Database for 2020. A multivariate logistic regression model was constructed with in-hospital mortality as the outcome variable and identifying predictor variables as defined by the Clinical Classifications Software Refined Variables (CCSR) codes for selected ICD-10 diagnoses. Descriptive statistics and the base logistic regression analyses were conducted using SAS statistical software version 9.4 (SAS Institute, Cary, NC, USA). To avoid overpowering, a subsample (*n *= 100,000) was randomly selected from the original dataset. The initial CCI assigned weights to ICD-10 diagnoses based on the associated risk of death, and conditions with the greatest collective weights were included in a subsequent backward stepwise logistic regression model.

Results

The results of the base CCI regression analysis revealed 16 chronic conditions with *P*-values <0.20. Anemia (1,567,081, 28.32%), pulmonary disease (asthma, chronic obstructive pulmonary disease [COPD], pneumoconiosis; 1,210,892, 21.88%), and diabetes without complications (1,077,239, 19.47%) were the three most prevalent conditions associated with inpatient mortality. Results of the backward stepwise regression analysis revealed that severe liver disease/hepatic failure (adjusted odds ratio [aOR] 10.50; 95% confidence interval [CI] 10.40-10.59), acute myocardial infarction (aOR 2.85; 95% CI 2.83-2.87) and malnutrition (aOR 2.15, 95% CI 2.14-2.16) were three most important risk factors and had the highest impact on inpatient mortality (*P*-value <0.0001). The concordance statistic (c-statistic) or the area under the curve (AUC) for the final model was 0.752.

Conclusions

The CCI model proved to be a valuable approach in categorizing morbidity classifications associated with the greatest risk of death using a national sample of hospitalized patients in 2020. Study findings provide an objective approach to compare patient populations that bear important implications for healthcare system improvements, clinician treatment approaches, and ultimately decision decision-makers poised to influence advanced models of care and prevention strategies that limit disease progression and improve patient outcomes.

## Introduction

This article gives insight into the inpatient mortality prediction and the comorbidity index. The article was previously presented as a meeting abstract at the 2023 Appalachian Research Forum Meeting on April 25, 2023.

Inpatient mortality represents an indicator of great significance to the United States both from a quality perspective [1,2] and an economic one. In-hospital mortality, as recognized by the United States Agency for Healthcare Quality (AHRQ) to represent the quality of care within hospitals, accounts for mortality based on three indicators: (1) select medical conditions and procedures; (2) procedures linked with questions of use (misuse and over/under use); and (3) high-volume procedures traditionally associated with lower mortality rates [1,3]. For a healthcare provider, knowing how the comorbidity models work for estimating in-hospital mortality is essential not only for determining patient health status in the hospital setting but also for regulating mortality risk and mortality risk predictions [4].

In terms of the economics of inpatient mortality, a team of researchers found that, according to estimates from 2007, the costs of hospitalization cases were 2.5 times higher for patients who died compared to those who were discharged. The stays that resulted in death comprised over 5% of all hospitalization costs that year, roughly translating into $17.6 billion of all hospital inpatient costs [3]. Therefore, continued examination of inpatient mortality is a heightened priority for the United States [5]. One of the most widely used discriminatory models is the Charlson model, which predicts the risk of mortality within one year of hospitalization of patients with various comorbidities using the Clinical Classifications Software Refined (CCSR) variables codes for the International Classification of Diseases, 10th Edition (ICD-10) diagnoses, which are quantified by the concordance statistics (c-statistics), represented by the area under the curve (AUC) [6]. The CCI model was developed by Dr. Mary Charlson and has been developed to be one of the most used models today. The CCI model assigns a score to each comorbidity based on its severity and potential impact on mortality. The score ranges from 1 to 6, with higher scores indicating a greater risk of mortality. The scores for each comorbidity are then added together to give a total score, which is used to predict the risk of mortality [7,8].

The Charlson model, as compared to other models such as the Elixhauser Comorbidity Index, is the preferred model by most healthcare professionals not only because it is an easy model to use and comprehensible but also a very reliable and valid method for predicting mortality and morbidity in our populations. One of the studies conducted by Charlson et al. revealed that the CCI model had good discriminative ability and validity to predict mortality in patients admitted with various chronic health conditions [9]. In other studies, the CCI is a powerful predictor of outcomes in a variety of settings, including surgical procedures, cancer treatment, and hospitalizations. For example, a study done by Piccirillo et al. found that CCI was a significant predictor of mortality in patients with advanced-stage lung cancer [10]. Despite being the most commonly used mortality predictor, some studies have found it to have some limitations. For example, the Charlson model only includes limited conditions on its applicability such as diabetes, heart disease, and cancer but conditions like mental status are not included as well as lack of consideration for age and gender which may influence the impact of comorbidity on patient outcomes during hospitalization. The Charlson Index assigns equal weight to each comorbidity, regardless of its severity or impact on patient outcomes [8].

This project aims to adapt a comorbidity index model to the National Inpatient Sample (NIS) database of 2020 to predict one-year mortality for patients admitted with chronic kidney diseases across different hospitals in the nation.

## Materials and methods

Data source 

The data analyzed in this study are based on data from the NIS for 2020. The NIS data are a part of the databases developed by the Healthcare Costs and Utilization Projects (HCUP), and it is the largest available inpatient database designed to produce the national estimates of inpatient costs, utilization, estimates, quality, and outcomes. The NIS database is available from 1988 to 2020, and the participating number of states has increased from 8 in the first year to 48 plus the District of Columbia currently [7]. The 2020 NIS data have the diagnoses and the procedure codes reported using the ICD-10 and Clinical Modification/Procedure Coding System (ICD-10-CM/PCS). The 2020 NIS data present approximately 98% of the US population containing a weighted population sample of 35 million hospitalizations nationally. Compared with the previous year’s database, the overall number of discharges for the data year 2020 decreased by 9% from 2019, while the inpatient mortality for this year increased from 2.0% to 2.8% [7]. The CCSR for the ICD-10-CM diagnoses aggregates more than 70,000 ICD-10-CM diagnosis codes into over 530 clinical categories across 22 body systems, and the CCSR for ICD-10-PCS procedures aggregates more than 80,000 ICD-10-PCS procedure codes into over 320 clinical categories across 31 clinical domains.

Study design and analysis

This is a retrospective analysis using the dataset obtained from the HCUP-NIS database. A Charlson comorbidity model based on the CCSR variables was adapted to obtain the mortality rate for a sample population of 5,533,477 aged 18 years or above in our dataset hospitalized with chronic diseases defined by the ICD-10-CM codes. All patients less than 18 years old were excluded from our study. To avoid overpowering, a subsample (*n *= 100,000) was randomly selected from the original dataset. The initial CCI assigned weights to ICD-10 diagnoses based on the associated risk of death, and conditions with the greatest collective weights were included in a subsequent backward stepwise logistic regression model (Table 1).

**Table 1:** Twenty-one CCSR-based variables. ^*^Excluded as predictors of inpatient mortality due to *P *> 0.2. ^**^Not included in the final model based on stepwise regression. CCSR, Clinical Classifications Software Refined

CCSR variables	CCSR codes
Acute myocardial infarction	CIR009
Congestive heart failure	CIR019
Perivascular disease	CIR026
Cerebral infarction	CIR020
Dementia/Neurocognitive disorder	NVS011
Pulmonary disease (asthma, COPD, and pneumoconiosis)	RSP008, RSP009, and RSP013
Liver disease	DIG019 and DIG02
Paralysis	NVS008
Renal disease	GEN003
Cancer	NEO001‐NEO069 and NEO071
Metastatic cancer	NEO070
Severe liver disease/liver failure	DIG018
Obesity	END009
Malnutrition	END008
Anemia**	BLD001-BLD005
Smoking	MBD024
Diabetes without complication	END002
Diabetes complications*	END003
Connective tissue disorder/rheumatologic*	MUS003, MUS008, and MUS024
Peptic ulcer disease*	DIG005
Human immunodeficiency virus*	INF006

Logistic regression analyses were conducted to obtain the final model using CCSR variables, as defined by the CCSR variables codes for selected ICD-10 diagnoses. Descriptive statistics and the base logistic regression analyses were conducted using SAS statistical software version 9.4 (SAS Institute, Cary, NC, USA). To avoid overpowering and variables attaining statistical significance while only marginally changing the outcome, a subsample (*n* = 100,000) was randomly selected from the original dataset. The subsequent backward stepwise logistic regression analysis included 20 CCSR variables with *P*-values <0.20 from the base simple logistic regression models. The multicollinearity among the included variables was not detected based on the variance inflation factor (VIF). The predictive power of the model was tested by the c-statistic or the AUC.

## Results

Tables 2-3 show the results of the final 16 CCSR variables model obtained. Logistic regression analysis was conducted to obtain the final model using 21 CCSR variables as dichotomous variables.

**Table 2:** results of the final 16 CCSR variables. Results of the final 16 CCSR variables model obtained and the characteristic distribution of the selected diagnosis in terms of total patients and the percentage. CCSR, Clinical Classifications Software Refined

Variables	Frequency (N)	Percentage (%)
Acute myocardial infarction		
Yes	248,852	4.5
No	5,284,625	95.5
Congestive heart failure		
Yes	1,039,785	18.79
No	4,493,692	81.21
Perivascular disease		
Yes	252,199	4.56
No	5,281,278	95.44
Cerebral disease		
Yes	154,111	2.79
No	5,379,366	97.21
Dementia		
Yes	425,377	7.69
No	5,108,100	92.31
Pulmonary disease		
Yes	1,210,892	21.88
No	4,322,585	78.12
Liver disease		
Yes	319,928	5.78
No	5,213,549	94.22
Paralysis		
Yes	131,209	2.37
No	5,402,268	97.63
Renal disease		
Yes	1,057,557	19.11
No	4,475,920	80.89
Cancer		
Yes	491,002	8.87
No	5,042,475	91.13
Metastasis		
Yes	190,918	3.45
No	5,342,559	96.55
Severe liver disease		
Yes	86,124	1.56
No	5,447,353	98.44
Obesity		
Yes	1,062,797	19.21
No	4,470,680	80.79
Malnutrition		
Yes	359,023	6.49
No	5,174,454	93.51
Smoking		
Yes	917,326	16.58
No	4,616,151	83.42
Diabetes without complication		
Yes	1,077,239	19.47
No	4,456,238	80.53

**Table 3:** Logistic regression analysis, OR, 95% CIs, and P-values. OR, odds ratio; CI, confidence interval

Variables	Adjusted OR	95% CI	*P*-value
Acute myocardial infarction	2.848	2.829-2.867	<0.0001
Congestive heart failure	1.677	1.668-1.685	<0.0001
Perivascular disease	1.154	1.143-1.164	<0.0001
Cerebral disease	2.128	2.105-2.151	<0.0001
Dementia	2.003	1.991-2.015	<0.0001
Pulmonary disease	1.197	1.191-1.203	<0.0001
Liver disease	0.797	0.790-0.804	<0.0001
Paralysis	1.39	1.372-1.407	<0.0001
Renal disease	1.428	1.420-1.436	<0.0001
Cancer	1.548	1.536-1.559	<0.0001
Metastasis	1.973	1.954-1.992	<0.0001
Severe liver disease	10.497	10.402-10.593	<0.0001
Obesity	0.807	0.803-0.812	<0.0001
Malnutrition	2.149	2.136-2.162	<0.0001
Smoking	0.504	0.500-0.508	<0.0001
Diabetes without complication	1.113	1.107-1.119	<0.0001

We excluded connective tissue/rheumatologic disorders, peptic ulcer disease, anemia, diabetes with complications, and human immunodeficiency as predictors of inpatient mortality.

The final model was conducted based on the original dataset. Anemia (1,567,081, 28.32%), pulmonary disease (asthma, COPD, and pneumoconiosis; 1,210,892, 21.88%), and diabetes without complications (1,077,239, 19.47%) were the three most prevalent conditions among hospitalized patients. Severe liver disease/hepatic failure (adjusted odds ratio [aOR] 10.50; 95% confidence interval [CI] 10.40-10.59), acute myocardial infarction (aOR 2.85; 95% CI 2.83-2.87) and malnutrition (aOR 2.15; 95% CI 2.14-2.16) were the three most important risk factors and had the highest impact on inpatient mortality. However, smoking history, obesity, and liver disease were negatively associated with inpatient mortality. The c-statistic or the AUC for the final model was 0.752.

## Discussion

Inpatient mortality remains an important outcome measure that is used to evaluate the quality of healthcare provided by hospitals and an improvement target for cost control efforts. Inpatient mortality rates can vary depending on factors such as patient age, severity of illness, comorbidities, and the hospital's level of resources and expertise [11-13]. Our study has revealed that different comorbidities are risk factors for mortality among hospitalized patients and that the mortality rate was among certain comorbidities and lower in others. It is important to mention that COVID-19 deaths may also have been a factor during the selected data year examined in this study [14].

The measurement of inpatient hospital mortality rates can be useful for identifying areas of improvement in hospital care and for comparing the quality of care provided by different hospitals [15]. However, it is important to note that inpatient mortality rates may not always be an accurate measure of the quality of care provided by a hospital, as some factors that contribute to mortality are outside the control of the hospital, such as the patient's underlying health conditions, Insurance status, and emerging disease trends [16,17].

Apart from the Charlson comorbidity index model, several other risk prediction models have been developed to predict inpatient hospital mortality, including the Acute Physiology and Chronic Health Evaluation (APACHE) score and the Simplified Acute Physiology Score (SAPS) [13]. These models use various patient characteristics and clinical variables to predict the likelihood of inpatient mortality and can be useful for identifying high-risk patients who may require more intensive care and monitoring [18].

This study has some limitations. First, NIS data lacked detailed clinical information. The NIS database contains limited clinical information on comorbidities, which may lead to the underreporting or misclassification of certain conditions. Second, this study included information only on hospitalizations, did not include data on outpatient care or primary care, which can impact the accuracy of the CCI calculation, and did not examine deaths by infectious disease/COVID status, immunization, gender, elective vs. emergency treatments, Medicaid vs. Medicare, uninsured status, or setting from which patients were hospitalized (from long-term care, rehabilitative facilities, or community settings) nor did it account for conditions of the patient (i.e., on chemotherapy, dialysis, or neurological conditions that may preclude malnutrition) [16]. Lastly, the study relied on diagnostic codes for identifying comorbidities, and there was a possibility of coding errors, which may lead to inaccurate CCI calculations.

Overall, from this study, we learned that the CCI model helps clinicians in different ways in decision-making. In clinical decision-making, the CCI can assist in determining the appropriate treatment plan. Patients with a high CCI score may require more cautious management and closer monitoring due to their increased vulnerability. The CCI can be used to predict outcomes such as mortality, length of hospital stays, and postoperative complications. This information helps healthcare providers and patients make informed decisions about treatment options and set realistic expectations.

## Conclusions

Our findings, based on Charlson modeling procedures, indicate that independent variables representative of comorbidity with the strongest one-year risk of mortality were among patients with ICD-10 codes relating to severe liver disease/hepatic failure, acute myocardial infarction, and malnutrition. Hence, relevant stakeholders (patients, family members, and healthcare providers) can utilize this knowledge to advance models of care and prevention strategies that limit disease progression and improve patient outcomes. While the CCI model is a valuable tool, it's important to consider that it's just one of several factors for assessing a patient's mortality. Factors such as age, gender, and the overall health status of the patient should also be taken into account. Overall, inpatient hospital mortality is an important outcome measure that can help identify areas of improvement in hospital care, ultimately improving patient outcomes and providing insights into cost containment avenues. However, it should be interpreted in the context of other factors that may influence patient mortality.
